# LONGITUDINAL CHANGE IN PERFORMANCE ON THE MONTREAL COGNITIVE ASSESSMENT IN THE PEOPLE WITH DEMENTIA

**DOI:** 10.1093/geroni/igad104.3458

**Published:** 2023-12-21

**Authors:** Kuan-Ching Wu, Jessica Jin, Shao-Yun Chien, Oleg Zaslavsky

**Affiliations:** University of Washington, Seattle, Washington, United States; University of Washington, Seattle, Washington, United States; University of Washington, Seattle, Washington, United States; University of Washington, Seattle, Washington, United States

## Abstract

The Montreal Cognitive Assessment (MoCA), a widely employed cognitive screening tool, is designed to identify mild cognitive impairment and diverse forms of dementia. Through its multidimensional evaluation of cognitive domains, MoCA supports clinicians in precise assessment and intervention planning for individuals manifesting cognitive deficits. Despite MoCA’s prevalence, limited research has explored its temporal dynamics in individuals experiencing memory loss, particularly across different stages of dementia. This study examined the MoCA changes of a longitudinal study analyzing the conversational speech in 8 older adults (5 males 3 females) with mild- to moderate-stage of dementia. Repeated biweekly measures at 12 intervals over six months were employed to gauge changes in total MoCA scores and its sub-tasks. Our findings reveal: 1) a significant negative correlation (r=-0.22, p=0.03) between the total MoCA score and the total time spent in tests; 2) most participants present a positive correlation (r=0.03 to 0.6) in their first memory trial session and a negative correlation (r=-0.1 to -0.8)in the second trial between words recalled and the time spent; 3) over 60% of participants demonstrating a declining trend in naming sessions, while orientation sessions exhibit no clear trend. These outcomes imply MoCA’s susceptibility to a “learning effect” with repeated measurements in people with dementia. Future longitudinal studies should consider alternate MoCA versions or extended assessment intervals to mitigate this effect.

